# Analysis and Compensation of Lorentz Force Magnetic Bearing Magnetic Flux Density Uniformity Error

**DOI:** 10.3390/s24092683

**Published:** 2024-04-24

**Authors:** Chunmiao Yu, Yuanwen Cai, Weijie Wang, Wenjing Han, Zengyuan Yin, Wenting Han

**Affiliations:** 1 The Department of Astronautics Science and Technology, Space Engineering University, Beijing 101416, Chinahwj_pll@163.com (W.H.); hwtcucurbit@163.com (W.H.); 2 The Astronaut Center of China, Beijing 100094, China; freeyzy1@163.com

**Keywords:** angular rate sensitivity, error compensation, Lorentz force magnetic bearing (LFMB), magnetic flux density, rotor tilt

## Abstract

Aiming at the influence of the magnetic flux density uniformity error (MFDUE) of the Lorentz force magnetic bearing (LFMB) on the sensitivity accuracy of magnetically suspended control and sensing gyroscopes (MSCSGs) on the angular rate of a spacecraft, a high precision measurement method of the angular rate of a spacecraft based on the MFDUE compensation of LFMB is proposed. Firstly, the structure of MSCSG and the sensitivity principle of MSCSG to the spacecraft angular rate are introduced. The mechanism influencing the accuracy of MSCSG to spacecraft angular rate sensitivity is deduced based on the definition of magnetic flux density uniformity. Secondly, the 3D magnetic flux distribution of LFMB is analyzed using ANSYS. The relationship between the rotor tilt angle, tilt angular rate, and magnetic flux density is established. The induced current calculation model due to MFDUE is proposed, and the LFMB magnetic flux density error compensation is realized. Finally, the simulation results show that the estimation accuracy of the induced current by the proposed method can reach 96%, and the simulation and the experiment show that the error compensation method can improve the accuracy of MSCSG in measuring the spacecraft angular rate by 12.5%.

## 1. Introduction

Magnetic bearings are playing an increasingly important role in the aerospace field because of their advantages, such as frictionless, long life, low energy consumption, and low noise [1,2,3]. Magnetically suspended control and sensing gyroscopes (MSCSGs) use magnetic bearings to control the five degrees of freedom (DOF) of magnetically suspended rotors [4,5,6]. MSCSGs have great development prospects in the field of spacecraft platform control due to their functions of attitude control and angular rate measurements at the same time [7,8,9].

The angular rate of MSCSGs can be calculated by detecting the current in the coils of Lorentz force magnetic bearings (LFMBs) so that the accuracy of LFMB design and machining directly affect the angular rate measurement accuracy of MSCSGs [10,11,12]. The compensation of the LFMB magnetic flux density uniformity error (MFDUE) is an effective way to improve the angular rate measurement accuracy of MSCSGs because the magnetic flux density uniformity (MFDU) of LFMB coils’ working air gap is the main factor affecting the current accuracy [13,14]. There is currently little research into the analysis and compensation of LFMB MFDUE. The main way to eliminate the error is to improve the magnetic circuit structure of LFMB. Yu [15,16] proposed a kind of LFMB for controlling the 2-DOF active tilt of a magnetically suspended inertia momentum wheel. However, the proposed structure had low air gap density uniformity and magnetic field intensity, which increased the energy consumption of LFMB. Xu [17] concluded that the MFDU of an LFMB working air gap is an important factor affecting the angular rate measurement accuracy of MSCSGs. He proposed a trapezoidal permanent magnet structure, which greatly improved the axial magnetic field intensity uniformity of the working air gap but did not solve the lack of circumferential MFDU caused by the block radial magnetization. Liu [18] proposed an internal LFMB to solve the problem of poor MFDU of an external LFMB and effectively attenuated the edge effect of a magnetic circuit at the end of a magnetic pole. Fu [19] proposed a spherical LFMB to achieve a larger angle tilt of a magnetically suspended rotor. It also improved the MFDU of an LFMB working air gap at the same time.

All the above methods improved the MFDU of an LFMB working air gap by optimizing the magnetic circuit structure of LFMB. However, the improvement level of MFDU is limited due to processing, assembly, temperature, and so on. So, it is necessary to put forward an appropriate MFDUE compensation method [20]. Xin [21] proposed an MFDUE compensation method for LFMB based on the Hall sensor probe online measurement. Based on an established model between the magnetic flux density and electromagnetic torque, the magnetic flux density measured by the Hall sensor was brought into the model to calculate the electromagnetic torque error to realize the magnetic flux density error compensation. But the addition of the Hall sensor can increase difficulty in prototype design. It is impossible to accurately estimate the magnetic flux density at the coil working place because of the limited magnetic flux density detection points. Based on the above analysis, this paper proposes an analysis and compensation of LFMB MFDUE with MSCSG as the object. This method establishes a model between magnetic flux density and the induced current, and then the magnetic flux density error compensation is realized by combining the relationship between magnetic flux density and the rotor tilt angle.

The rest of this paper is as follows. The structure of MSCSG and the angular rate sensitivity principle are introduced in Section 2. The relationship between the induced current in the coil and the magnetic flux density of the LFMB working air gap is derived. In Section 3, the induced current calculation model due to MFDUE is proposed based on a simulation analysis of the LFMB 3D magnetic field. Then, a sensitivity model of the spacecraft angular rate compensated by MFDUE is obtained. Section 4 verifies the estimation accuracy of the induced current and the improving effect of MSCSG on spacecraft angular rate measurement accuracy by magnetic flux density error compensation through simulation and experiment. Finally, it draws the conclusion of this paper in Section 5.

## 2. LFMB Structure and Error Analysis

### 2.1. MSCSG Structure

**Figure 1:**
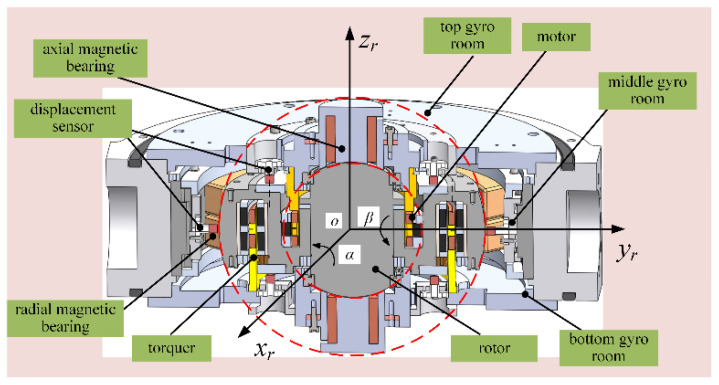
RLFMB control load compartment/frame compartment deflection.

A 3D diagram of the MSCSG structure is shown in Figure 1. It is mainly composed of magnetic bearings, a motor, displacement sensors, a rotor, and gyro rooms. Among them, the magnetic bearings can apply magnetic suspended force to the rotor to ensure its 5-DOF stable control. The motor is used to drive the rotor to rotate at high speed. The displacement sensors are used to detect the position and attitude information of the rotor. The gyro rooms are used to fix the supporting stator components.

**Figure 2:**
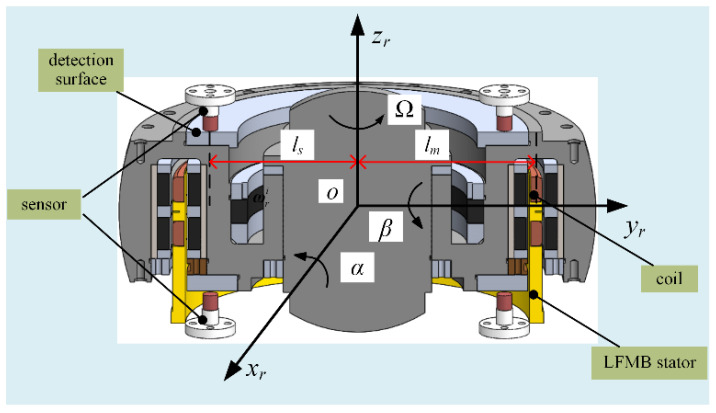
Schematic diagram of MSCSG rotor structure.

Next, the control principle of the MSCSG rotor is briefly introduced. Figure 2 shows the 3D structure of the rotor. Under normal working conditions, the rotor rotates around the *z_r_* axis at a high speed of Ω. When the attitude of MSCSG is changed, the axial direction of the rotor remains unchanged due to the fixed axis of the gyroscope, and the displacement sensor can detect that the position of the rotor changes relative to the stator. The rotor tilt displacement signal is fed back to the controller through a signal conditioning circuit and the controller issues instructions to change the current of the LFMB coils to control the rotor to follow the stator movement.

### 2.2. LFMB Structure and Sensitivity Principle

**Figure 3:**
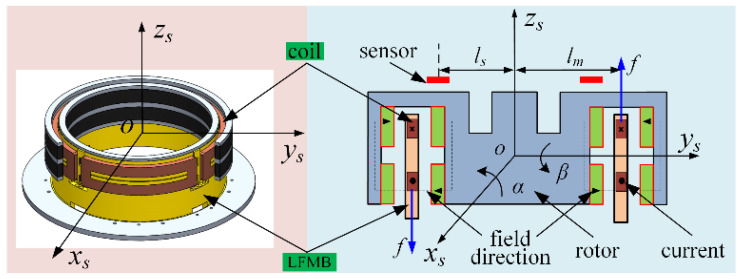
Schematic diagram of LFMB structure.

LFMB, a kind of magnetic bearing that controls rotor deflection around the 2-DOF radial direction, is the core component of MSCSG to realize its main function. According to the characteristic that the output torque is proportional to the control current, MSCSG can detect spacecraft attitude with high precision. The structure of LFMB is shown in Figure 3, where the material of the permanent magnet is samarium–cobalt alloy, and the material of the coil skeleton is machinable ceramics. The relationship model between its output torque ***M_g_*** and current can be expressed as

(1) $$M_{g}=\left(\begin{array}{c} M_{gx}\\ M_{gy} \end{array}\right)=l_{m}\times K_{I}I=\left(\begin{array}{c} 4NBLl_{m}I_{\alpha }\\ -4NBLl_{m}I_{\beta } \end{array}\right)$$

where $M_{gx}$ and $M_{gy}$ represent torques applied by LFMB to the rotor in different directions, respectively; *l_m_*, *K_I_*, *N*, *B*, and *L* represent the radius of the LFMB stator, current stiffness, the number of the coil turns, the magnetic flux density of the working air gap, and the effective length of the coil, respectively, where *K_I_* = 4*NBL*; and $I_{\alpha }$ and $I_{\beta }$ represent the control current through the LFMB.

MSCSG controls the active tilt of the rotor to change the direction of the angular momentum to output control torque to the spacecraft. At the same time, the angular rate information of the spacecraft with two degrees of freedom can be calculated by detecting the control current in the LFMB coil and combining the angular motion relationship between the spacecraft and MSCSG. The sensitivity formula of a single machine MSCSG can be calculated as [22]

(2) $$\left\{ \begin{array}{l} \dot{\delta }=\frac{-2\sqrt{2}NBLl_{m}(I_{\alpha }-I_{\beta })+\frac{\sqrt{2}}{2}(J_{r}(\ddot{\alpha }+\ddot{\beta })-J_{z}\Omega (\dot{\alpha }-\dot{\beta }))}{J_{z}\Omega }\\ \dot{\theta }=\frac{2\sqrt{2}NBLl_{m}(I_{\alpha }+I_{\beta })-\frac{\sqrt{2}}{2}(J_{r}(\ddot{\alpha }-\ddot{\beta })+J_{z}\Omega (\dot{\alpha }+\dot{\beta }))}{J_{z}\Omega } \end{array}\right.$$

where $\dot{\delta }$ and $\dot{\theta }$ represent the carrier angular rate information, respectively. $J_{r}$ and $J_{z}$ represent the radial and axial moment of inertia of the magnetic suspension rotor, respectively; Ω represents the speed of rotor; $\dot{\alpha }$ and $\dot{\beta }$ represent the rotor 2-DOF deflection angular rate calculated from the detection information of the displacement sensor; $\ddot{\alpha }$ and $\ddot{\beta }$ represent the corresponding angular acceleration of the rotor.

### 2.3. LFMB Magnetic Flux Density Error Analysis

As shown in Figure 3, the four coils of LFMB are installed on the LFMB stator skeleton. The upper, lower, inner, and outer four groups of annular magnetic steel are installed on the rotor. Each group of magnetic steel is made of small pieces of magnetic steel. The magnetic flux density near the LFMB coil can change with the change in coil position due to the splicing of the magnetic steel and the edge effect of the magnetic circuit. The variation degree in magnetic flux density is a quantifiable error, which is defined as MFDU as follows

(3) $$\xi =1-\frac{\sqrt{\frac{1}{n}\sum_{i=1}^{n}{\left(\bar{B}-B_{i}\right)}^{2}}}{\bar{B}}$$

where ξ represents the uniformity of magnetic flux density; $B_{i}$ and $\bar{B}$ represent the magnetic flux density of the *i*-th point at the coil and its mean value, respectively. ξ=1 when the magnetic flux density is completely uniform. The closer ξ is to zero, the worse MFDU is.

MFDU at the LFMB coil can be divided into the circumferential and axial magnetic flux density uniformity of the rotor. The rotor is running at high speed and stationary due to being installed on the stator. The magnetic steel rotates with the rotor at high speed; that is, the LFMB coil only has rotation relative motion around the rotor axis. At this time, the magnetic flux density at the coil changes with time due to the uneven circumferential magnetic flux density of the LFMB working air gap, so the magnetic flux passing through the coil changes at all times. According to Faraday’s law of electromagnetic induction, the coil can generate an induced electromotive force when the magnetic flux through the closed coil changes. The electromotive force due to the uniformity of circumferential magnetic flux density can be obtained as

(4) $$e_{c}=N\frac{\Delta \Phi }{\Delta t}=N\frac{\Delta BS}{\Delta t}=N\dot{B}S$$

where *e_c_* represents the electromotive force generated in the LFMB coil due to the change in magnetic flux; ΔΦ represents the change in magnetic flux passing through the LFMB coil over a period of time; Δt represents the elapsed time. ΔB represents the magnetic flux density variance at the coil; $\dot{B}$ represents the change rate of the LFMB coil at the current time; S represents the area perpendicular to the direction of the closed coil and the magnetic field.

**Figure 4:**
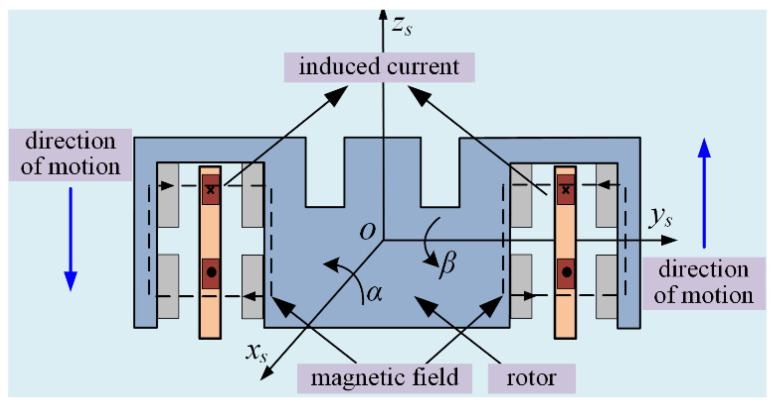
Schematic diagram of induced current direction generated by axial MFDU.

As shown in Figure 4, the coil can be regarded as the upper and lower wires cutting magnetic lines of motion. When the rotor is deflected, the two wires move in the same direction with the magnetic field. Since the electromotive force generated on the two wires is in opposite directions, the addition of the electromotive force generated by the two wires is the electromotive force generated in the coil due to the uniformity of axial magnetic flux density. We can obtain

(5) $$e_{a}=NLv\left(B_{2}-B_{1}\right)$$

where $B_{1}$ and $B_{2}$ represent the magnetic flux density near the upper and lower wires, respectively. *v* represents the moving speed of the coil relative to the stator. Thus, the induced current generated by the circumferential and axial MFDU in the LFMB coil is expressed as

(6) $$i=i_{a}+i_{c}=\frac{e_{a}+e_{c}}{R}=\frac{N}{R}\left(\dot{B}S+LV(B_{2}-B_{1})\right)$$

where $i_{c}$ and $i_{a}$ represent the induced current generated by the circumferential and axial MFDU of LFMB, respectively; *i* represents the total induced current generated.

## 3. LFMB Magnetic Flux Density Simulation Analysis and Error Compensation

### 3.1. Simulation Analysis of LFMB Magnetic Field

**Figure 5:**
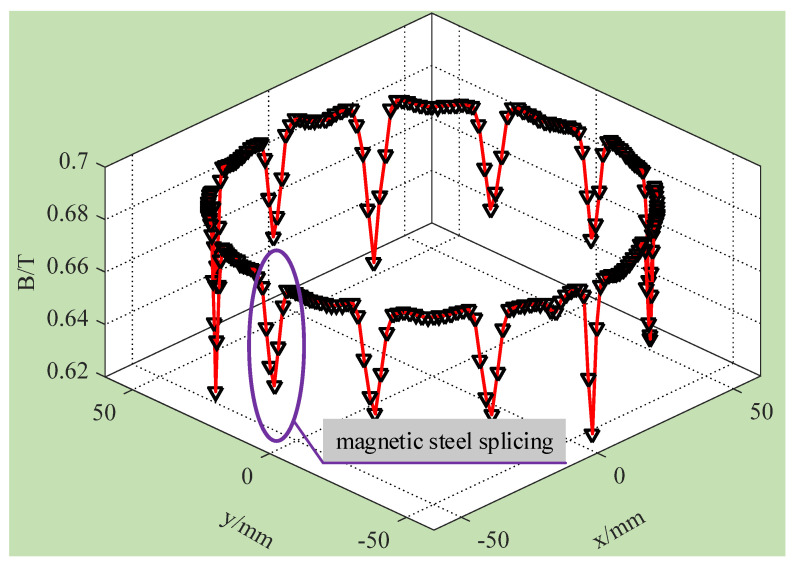
Magnetic field 3D distribution of LFMB working air gap circumferential.

Since it is difficult to achieve radial magnetization of the whole-ring magnetic steel with the current process, LFMB magnetic steel adopts splicing mode. Each small piece of magnetic steel is magnetized along the radial direction and then spliced to make the spliced magnetic steel form a closed-ring magnetic circuit. In this way, the circumferential uniformity of the LFMB working air gap magnetic flux density can be reduced. As shown in Figure 5, the magnetic flux density is greatly weakened at the magnetic steel splicing, which reduces the uniformity of circumferential magnetic flux density. Due to the low circumferential MFDU of the LFMB working air gap, the magnetic flux passing through the LFMB coil can change at any time when the rotor rotates at a high speed, resulting in an induced current. It seriously affects the sensitivity accuracy of MSCSG.

**Figure 6:**
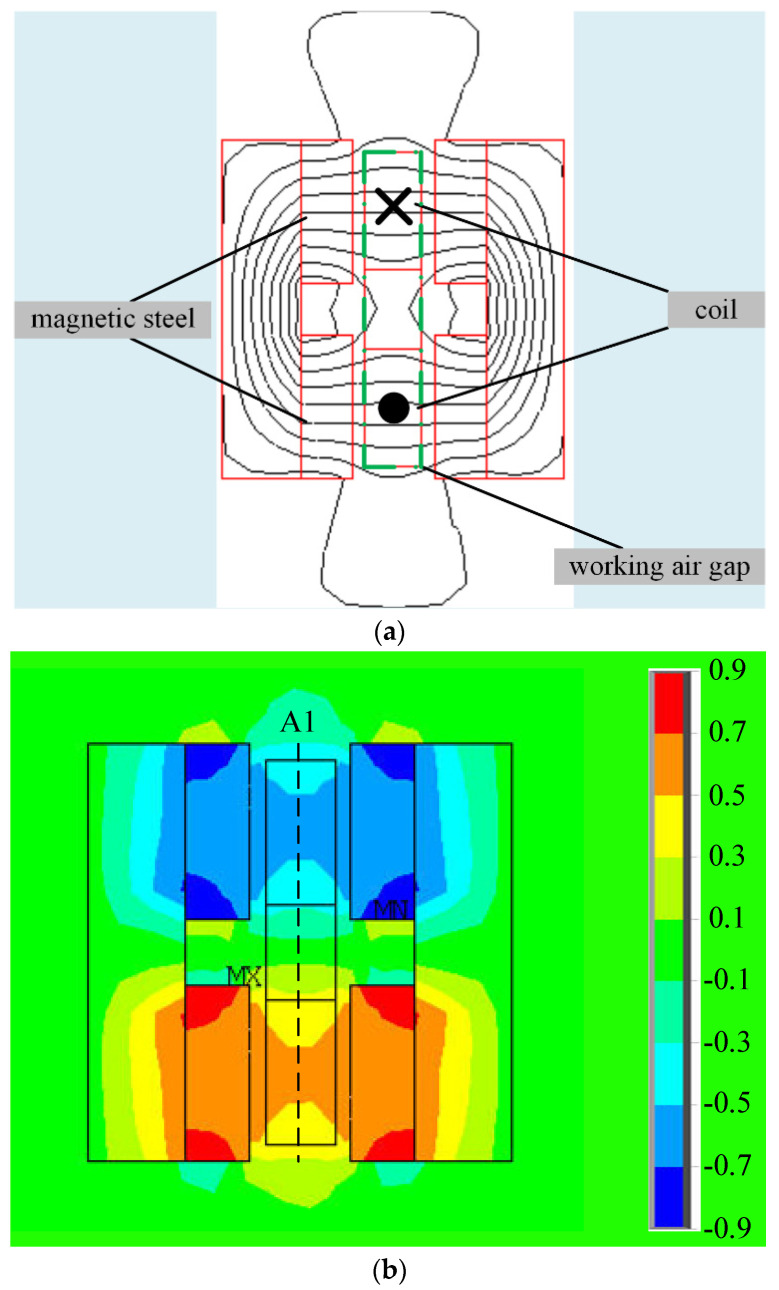
Magnetic field 2D distribution of LFMB working air gap circumferential: (**a**) the magnetic flux density line; (**b**) the radial component of the magnetic field intensity.

**Figure 7:**
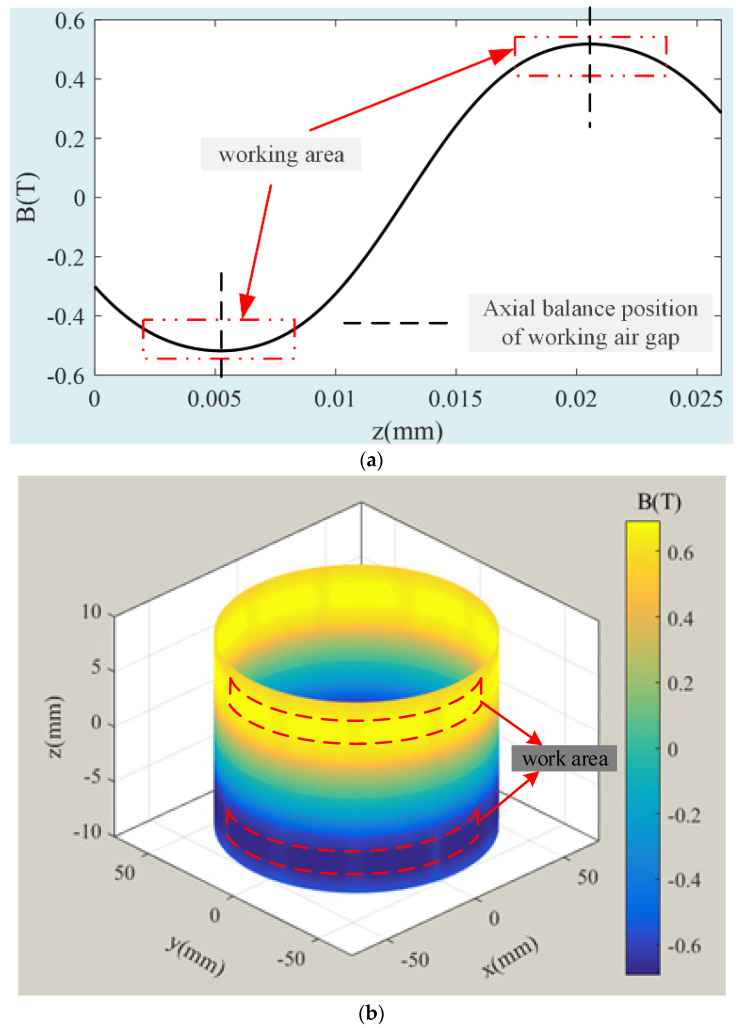
Magnetic flux distribution diagram at radial equilibrium position of LFMB working air gap: (**a**) path A1 2D magnetic flux density; (**b**) 3D magnetic flux distribution at radial equilibrium position of LFMB working air gap.

Figure 6 shows the 2D and 3D distribution of LFMB magnetic field intensity. It can be seen from Figure 6a that the magnetic flux density line at the LFMB coil changes when the rotor deflects. The overall distribution trend of the radial component of the magnetic field intensity at the LFMB working air gap is shown in Figure 6b. The magnetic flux density along the axial direction of the magnetic steel is larger and more uniform, and the farther the axial distance from the magnetic steel, the smaller the magnetic flux density. To more intuitively display the magnetic field intensity distribution of the LFMB working air gap, the A1 path (LFMB working air gap radial equilibrium position) in Figure 6b is taken as the magnetic flux density analysis path, and the 2D magnetic flux density analysis diagram in Figure 7a is obtained. The abscissa position to the dotted line position is the axial equilibrium position of the LFMB working air gap. With the rotor axis as the center, circles perpendicular to the rotor axis are drawn at different heights in the A1 path in Figure 6b as magnetic flux density analysis paths. Finally, the 3D magnetic flux distribution diagram of the radial equilibrium position of the LFMB working air gap is obtained, as shown in Figure 7a.

Figure 7a shows the axial distribution of magnetic flux density at the LFMB working air gap intuitively. The magnetic flux density is the largest at the axial equilibrium position, and it decreases slightly away from the equilibrium position, approximately presenting a sinusoidal distribution. Figure 7b shows that the magnetic flux density of the upper and lower parts of the LFMB working air gap is symmetrically distributed with opposite signs.

### 3.2. LFMB Magnetic Flux Density Error Compensation

The uneven magnetic flux distribution of the LFMB working air gap leads to the constant change in magnetic flux passing through the coil, and then the induced current generated by Equation (8) affects the sensitivity accuracy of MSCSG. The LFMB ring magnetic steel is composed of 12 pieces of small magnetic steel splicing, and the change rule of magnetic flux density at the splicing is similar, so the LFMB coil can produce 12 times the induced current in every revolution of the rotor. The rotor speed is 5000 r/min under the normal working condition of MSCSG, so the frequency of the induced current can reach 5000 r/min ÷ 60 s/min × 12 r^−1^ = 1000 Hz. The bandwidths of output torque and the sensitivity of MSCSG are 330 Hz and 140 Hz, respectively, both much lower than 1000 Hz. Therefore, this paper intends to eliminate the disturbance caused by circumferential MFDU to the LFMB coil current by a low-pass filter.

**Figure 8:**
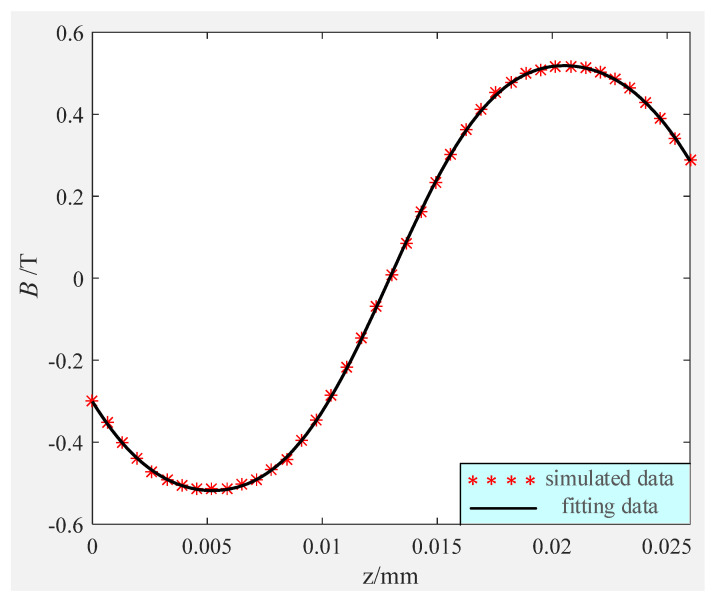
Fitting results of axial magnetic flux distribution of LFMB working air gap.

The axial magnetic flux distribution of the LFMB working air gap is relatively regular. By fitting the curve in Figure 7a, the curve formula fitted in Figure 8 can be obtained as follows

(7) $$B_{a}(z_{a})=0.542\sin(201.6z_{a}-2.605)-0.0242\sin(587.5z_{a}-4.495)$$

where *z_a_* represents the axial displacement of the coil relative to the stator; *B_a_*(*z_a_*) represents the magnetic flux density of the coil at *z_a_*. According to the LFMB structure, the axial coordinate of the upper part of the coil is 20.5 mm, and the axial coordinate of the lower part of the coil is 5.5 mm. When the rotor is in the balance position, the magnetic flux densities of the upper and lower parts of the coil are *B_a_*(20.5) = 0.5183 T and *B_a_*(5.5) = −0.5173 T, respectively.

*z_a_* and ${\dot{z}}_{a}$ can be obtained from the signal *h_α_* of rotor axial displacement sensor as

(8) $$z_{a}=\frac{l_{m}}{l_{s}}h_{\alpha },{\dot{z}}_{a}=\frac{l_{m}}{l_{s}}{\dot{h}}_{\alpha }$$

where *l_m_* and *l_s_* are the installation radii of the LFMB coil and axial displacement sensor, respectively. When the axial displacement sensor detects that the tilt displacement of the rotor is *h_α_*_1_ and the tilt line speed is ${\dot{h}}_{\alpha 1}$, the induced electromotive force and current generated by the upper and lower wires of LFMB can be obtained from (4). It can be judged that the electromotive force generated by the two wires is in the same direction according to the right-hand rule. Finally, by combining (4), (7), and (8), it can be obtained that the induced current *i_c_* generated by the axial MFDU is

(9) $$i_{c}=\frac{e_{c}}{R}=\frac{N}{R}\left(B_{a}(0.0205+\frac{l_{m}h_{\alpha }}{l_{s}})-B_{a}(0.0055+\frac{l_{m}h_{\alpha }}{l_{s}})\right)\frac{l_{m}{\dot{h}}_{\alpha }L}{l_{s}}$$

Finally, the sensitivity model of MSCSG on the angular rate of spacecraft after magnetic flux density compensation can be written as

(10) $$\left\{ \begin{array}{l} \dot{\delta }=\frac{-2\sqrt{2}NBLl_{m}\left(\left(I_{\alpha }-i_{\alpha })-(I_{\beta }-i_{\beta }\right)\right)+\frac{\sqrt{2}}{2}(J_{r}(\ddot{\alpha }+\ddot{\beta })-J_{z}\Omega (\dot{\alpha }-\dot{\beta }))}{J_{z}\Omega }\\ \dot{\theta }=\frac{2\sqrt{2}NBLl_{m}\left(\left(I_{\alpha }-i_{\alpha })+(I_{\beta }-i_{\beta }\right)\right)-\frac{\sqrt{2}}{2}(J_{r}(\ddot{\alpha }-\ddot{\beta })+J_{z}\Omega (\dot{\alpha }+\dot{\beta }))}{J_{z}\Omega } \end{array}\right.$$

where $i_{\alpha }$ and $i_{\beta }$ represent the induced currents generated in the *y_r_*-axis and *x_r_*-axis coils due to the MFDUE of LFMB, respectively.

**Figure 9:**
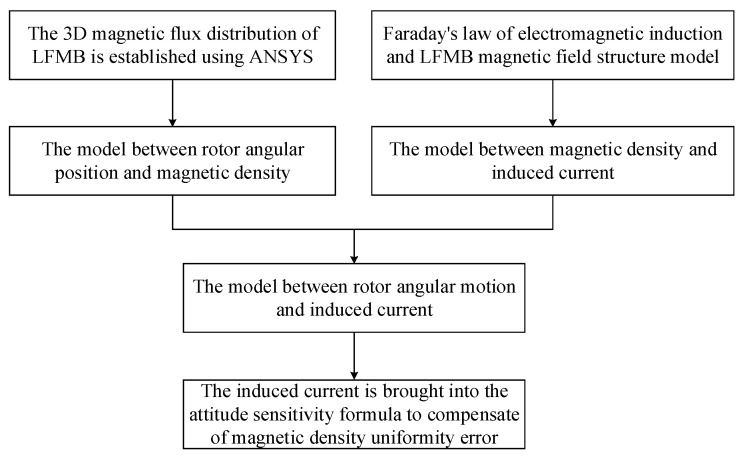
Analysis and compensation process chart of MFDUE of LFMB.

Thus, the magnetic density uniformity error compensation of LFMB is realized, and the overall compensation process is shown in Figure 9.

## 4. Simulation and Experimental Results

### 4.1. Simulation Verification of Compensation Method

**Figure 10:**
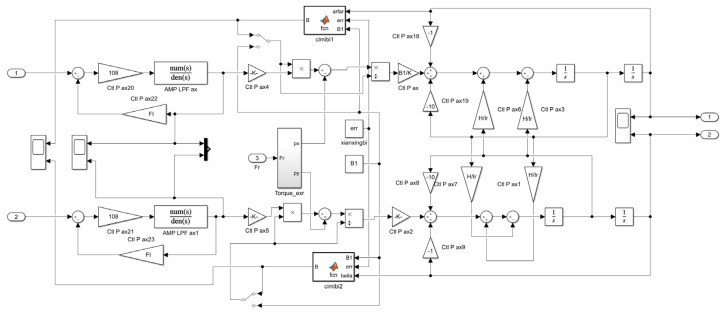
The core part of the MSCSG prototype simulation model.

The MSCSG prototype is modeled by Simulink to verify the accuracy of the induced current model and the compensation effect for the measurement accuracy of the gyro angular rate. Some simulation parameters are listed in Table 1. The core part of the MSCSG prototype simulation model is shown in Figure 10.

**Figure 11:**
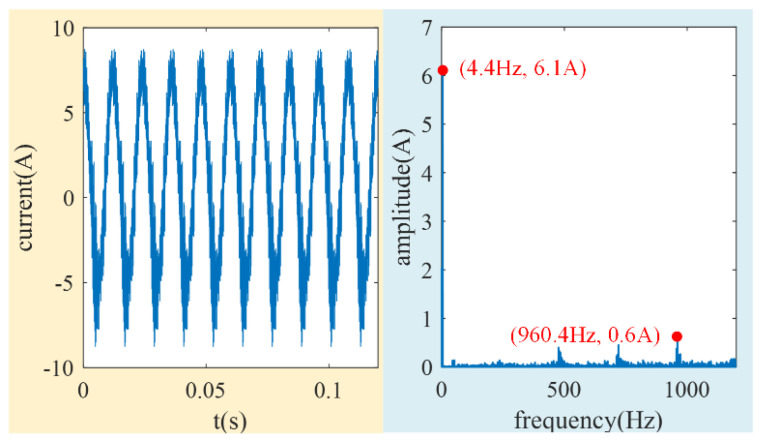
Schematic diagram of reference induced current and its spectrum.

Firstly, the accuracy of the induced current calculation model proposed in this paper is verified. The simulation model of the LFMB coil induced current is established by the ANSYS Electronics Desktop simulation program, and the calculation results of the model are taken as the reference. The rated speed of the MSCSG rotor is 5000 r/min (about 83.3 Hz), and the maximum rotor tilt angle is 0.3° (about 5.24 × 10^−3^ rad). Therefore, the rotor tilt angle amplitude is set as 5.24 × 10^−3^ rad, and the frequency of the rotor speed is set as 83.3 Hz. The induced current obtained after running the program is used as the reference induced current. Its simulation results and spectrum analysis are shown in Figure 11.

**Figure 12:**
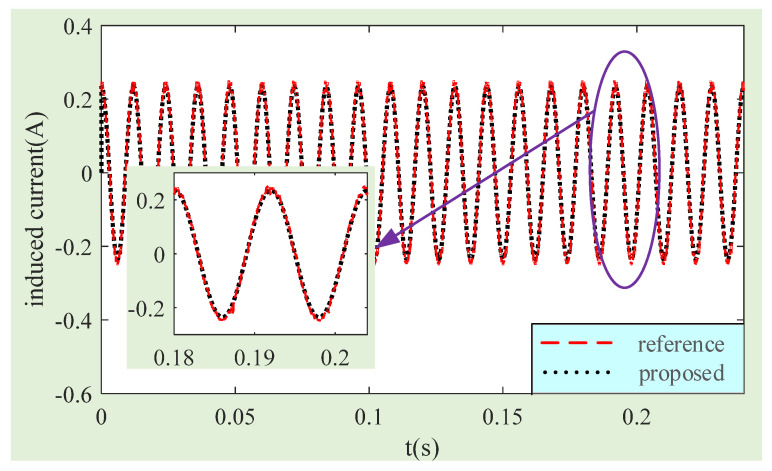
Comparison curves of the reference current and the induced current calculated by the proposed method.

It can be seen from the above analysis that the high-frequency component is mainly generated by the circumferential MFDUE of the LFMB working air gap. This part of the signal can be easily removed by the low-pass filter in the MSCSG control circuit for the MSCSG prototype. The amplitude of the simulated high-frequency component is one order of magnitude lower than that of the main frequency, as shown in Figure 11. The reference induced current is processed by sliding smoothing to achieve the effect of low-pass filtering and ensure that the result of the simulation is close to reality. Then, the rotor tilt angle and speed frequency in the simulation conditions are substituted into (11) to obtain the induced current calculated by the method proposed in this paper. In Figure 12, it shows the comparison between the processed reference induced current and that calculated by the method proposed in this paper. The amplitude of the reference induced current and the current obtained by the proposed method are 0.246 A and 0.235 A, respectively. That is, the estimation accuracy of the induced current by the proposed method can reach 96%. The accuracy of the induced current calculation method for the MFDUE is verified.

**Figure 13:**
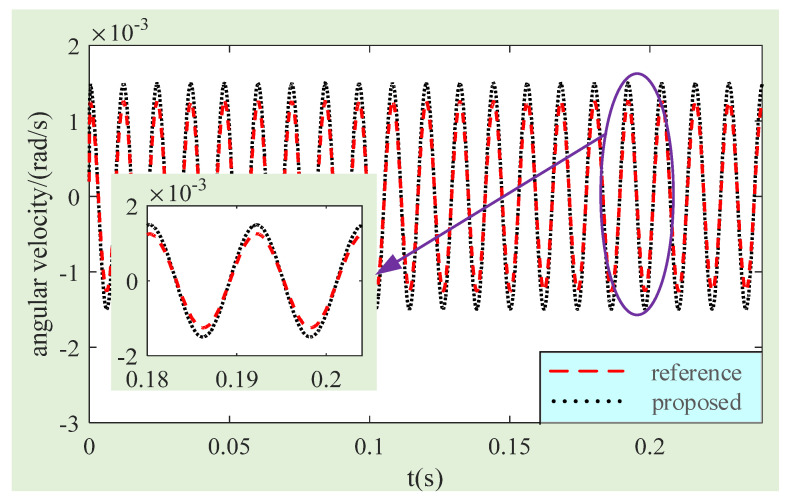
Comparison curves of gyro angular rate simulation results before and after magnetic flux density compensation.

Then, the calculation results of MSCSG regarding the spacecraft angular rate before and after magnetic flux density compensation are compared to verify the effectiveness of the compensation method in improving measurement accuracy. In the simulation model, the amplitude of the rotor tilt is set as 5 × 10^−4^ rad, the frequency of rotor speed is 83.3 Hz, and the angular rate of the spacecraft is set as earth speed (about 7.27 × 10^−5^ rad/s). The simulation results obtained are shown in Figure 13, in which two lines represent the calculated results of the MSCSG angle rate before and after magnetic flux density compensation, respectively. After calculation, the standard deviation of MSCSG to the spacecraft angular rate calculated before compensation is 1.058 × 10^−3^ rad/s, and the standard deviation of the angular rate calculated after magnetic flux density compensation is 8.856 × 10^−4^ rad/s. Therefore, the accuracy of MSCSG in measuring the spacecraft angular rate can be improved by about 16.3% after magnetic flux density compensation. It can be concluded that the MFDUE compensation method of LFMB proposed in this paper can improve the accuracy of MSCSGs in measuring spacecraft angular rates.

### 4.2. Experimental Verification of Compensation Method

**Figure 14:**
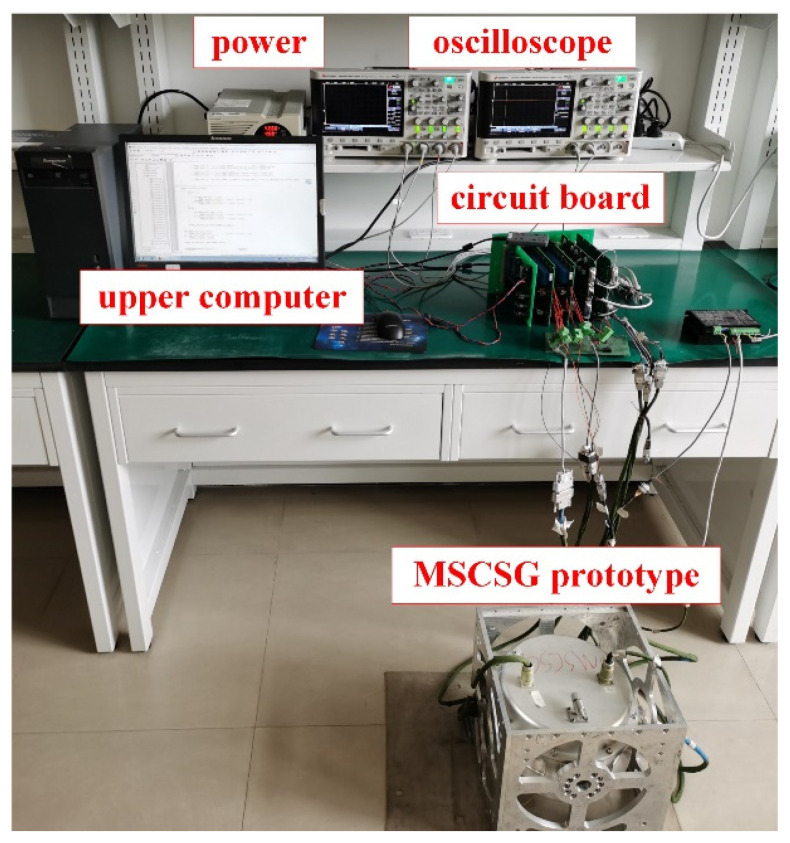
MSCSG experimental device.

Next, the feasibility of the proposed method can be verified with the MSCSG prototype developed by the research group. Some parameters of the MSCSG prototype are shown in Table 1. The experimental devices mainly include the MSCSG prototype, circuit board, upper computer, power, and oscilloscope, as shown in Figure 14.

**Figure 15:**
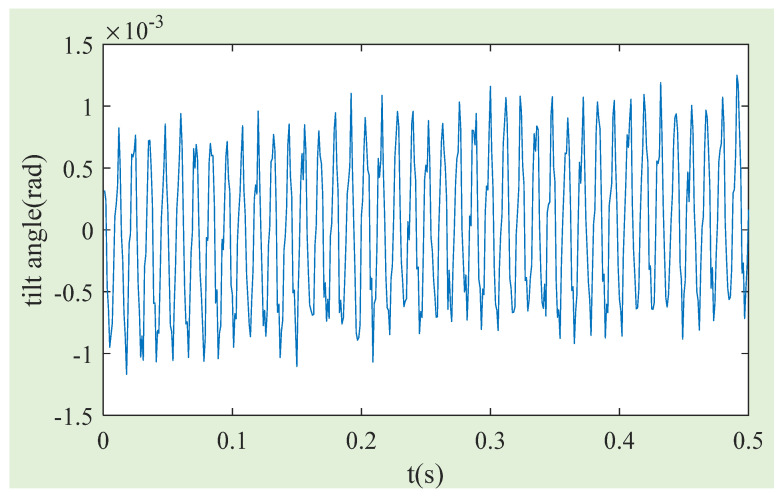
MSCSG rotor tilt angle at 5000 r/min.

Then, the feasibility of the compensation method proposed in this paper to improve the accuracy of MSCSG in measuring the spacecraft angular rate is verified by comparing the measurement results of MSCSG of the spacecraft angular rate before and after magnetic flux density compensation. Figure 15 shows the information on the rotor tilt angle collected when the MSCSG rotor runs at 5000 r/min.

**Figure 16:**
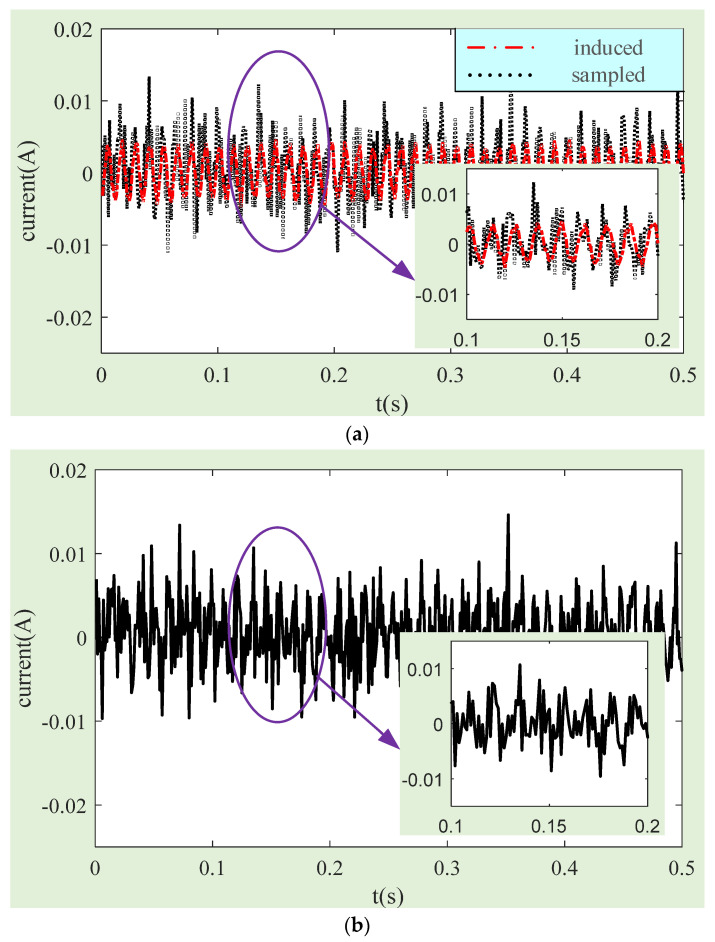
Comparison curves of the collected current and the induced current: (**a**) the collected current and calculated induced current; (**b**) magnetic flux density compensated current.

According to this information, the rotor tilt angle and tilt angular rate can be obtained, which can be substituted into (11) to obtain the induced current caused by the axial magnetic uniformity error. Figure 16a shows a comparison between the calculated induced current and the current collected in the experiment. The induced current calculated is the disturbance current caused by the magnetic flux density error of the LFMB coil working air gap. The current compensated by magnetic flux density can be obtained by calculating the difference between the experimental collected current and the induced current. The compensated current is shown in Figure 16b.

**Figure 17:**
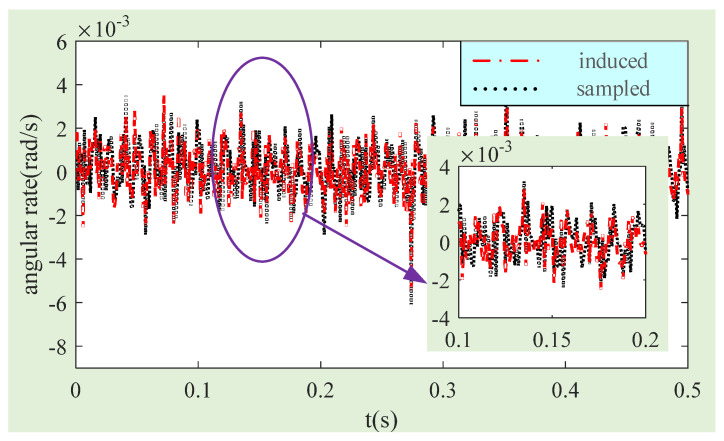
Experimental comparison curves of angular rate measurement before and after magnetic flux density compensation.

The angular rate of MSCSG is calculated for the experimentally collected current and compensated current, respectively. The comparison results are obtained, as shown in Figure 17. After calculation, the standard deviation of the MSCSG angle rate is 1.28 × 10^−3^ rad/s by the current before magnetic flux density compensation, and it is 1.12 × 10^−3^ rad/s by the current after compensation. Therefore, after magnetic flux density compensation, the measurement accuracy of MSCSG of the spacecraft angular rate can be improved by about 12.5%.

## 5. Conclusions

LFMB controls the tilt 2-DOF of the MSCSG rotor. The induced current can be generated in the coil due to MFDUE at the LFMB working air gap, thus reducing the measurement accuracy of MSCSG of the spacecraft angular rate. In this paper, the relationship between the induced current and rotor tilt angle motion is accurately deduced by establishing an LFMB magnetic flux density error model. Through simulation verification, the accuracy of the relationship can reach 96%.

Through simulation and experiment, the measurement accuracy of MSCSG of the spacecraft angular rate can be improved by 12.5% after magnetic flux density compensation. It can be said that magnetic flux density compensation can effectively reduce the influence of rotor tilt disturbance and active tilt on the measurement accuracy of MSCSG of the spacecraft angular rate. Therefore, the method proposed in this paper can effectively improve the contradiction between the precision of MSCSG output torque and the angular rate measurement.

## Figures and Tables

**Table 1:** MSCSG system simulation parameters.

Symbol	Quantity	Value
*J_r_*	Transverse moments of inertia	0.0097 kg·m^2^
*J_z_*	Polar moments of inertia	0.0167 kg·m^2^
*L*	Circumferential length of the coil	80 mm
*N*	Coil turns	200
Ω	Rotor speed	5000 r/min
*l_m_*	Radius of LFMB stator	59 mm
*l_s_*	Radius of LFMB coil	48.5 mm

## Data Availability

Data are contained within the article.
